# Supplementary material for: Distinct skyrmion phases at room temperature in two-dimensional ferromagnet Fe3GaTe2
Source: Nat Commun. 2024 Apr 16;15:3278. doi: 10.1038/s41467-024-47579-9 (PMC11021542; doi:10.1038/s41467-024-47579-9)
Supplement: Supplementary file 1 — Supplementary Information [file 41467_2024_47579_MOESM1_ESM.pdf]

Supplementary Information for

Distinct Skyrmion Phases at Room Temperature in Two-dimensional Ferromagnet Fe_3GaTe_2

Xiaowei Lv[1,6], Hualiang Lv[2,6], Yalei Huang[3,6], Ruixuan Zhang[4], Guanhua Qin[4], Yihui Dong[3], Min Liu[1], Ke Pei[1], Guixin Cao[3,4,✉], Jincang Zhang[4], Yuxiang Lai[5], Renchao Che[1,4,✉]

[1]X. Lv, M. Liu, K. Pei, Prof. R. Che
Laboratory of Advanced Materials, Shanghai Key Lab of Molecular Catalysis and Innovative Materials, Academy for Engineering & Technology, Fudan University, Shanghai 200438, P. R. China

[2]H. Lv
Shanghai Frontiers Science Research Base of Intelligent Optoelectronics and Perception, Institute of Optoelectronics, Fudan University, Shanghai, 200433 P. R. China

[3]Y. Huang, Y. Dong, Prof. G. Cao
Materials Genome Institute, Shanghai University, Shanghai 200444, China

[4]R. Zhang, G. Qin, Prof. G. Cao, J. Zhang, Prof. R. Che
Zhejiang Laboratory, Hangzhou 311100, China

[5]Prof. Y. Lai
Pico Electron Microscopy Center, Innovation Institute for Ocean Materials Characterization, Center for Advanced Studies in Precision Instruments, Hainan University, Haikou 570228, China

[6]These authors contributed equally: Xiaowei Lv, Hualiang Lv, Yalei Huang.

✉Corresponding authors. E-mail: rcche@fudan.edu.cn and guixincao@shu.edu.cn

This file includes:

Fig. S1 Energy-dispersive X-ray (EDX) results.

Fig. S2 Temperature dependence of magnetization curves with a magnetic field of 1000 and 10000 Oe.

Fig. S3 Temperature-dependent magnetic anisotropy.

Fig. S4 Thickness measurements of a uniform FGT lamella.

Fig. S5 Tilt-dependent contrast of magnetic domain.

Fig. S6 Evolution of magnetic domain structures in a FGT lamella.

Fig. S7 The schematic diagram for FC procedure.

Fig. S8 FC-dependent magnetic domain

Fig. S9 Thickness measurements of a FGT lamella with two different-thickness regions.

Fig. S10 The thermostability of the magnetic domain in the 114 nm-thick region.

Fig. S11 The thermostability of the magnetic domain in the 171 nm-thick region.

Fig. S12 Transport properties of FGT nanoflake.

Fig. S13 Micromagnetic simulations and theoretical LTEM images.

Fig. S14 The analysis of crystal structure of FGT.

Fig. S15 The results of first-principles calculations. a Schematic of DMI in FGT.

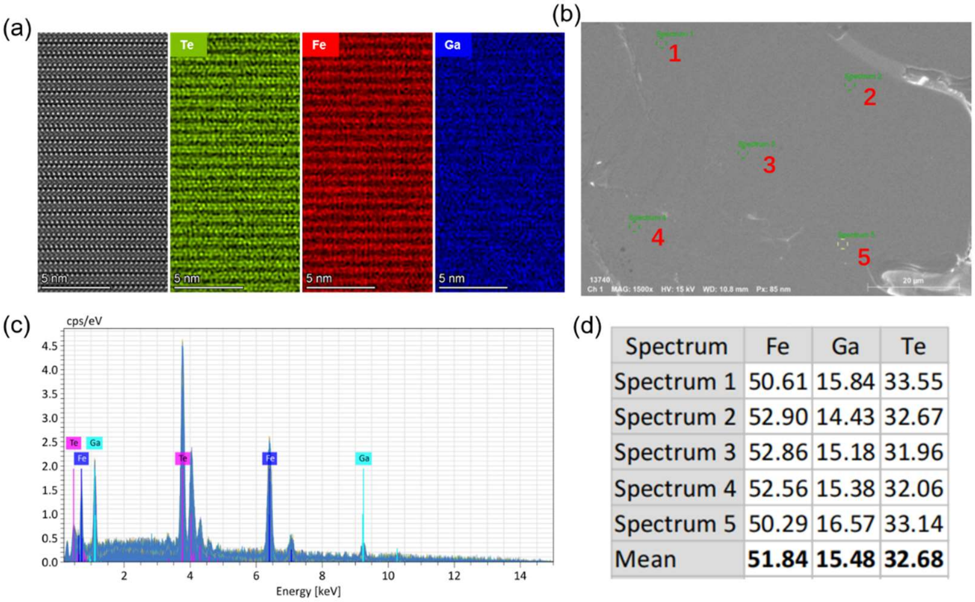

Spectrum	Fe	Ga	Te
Spectrum 1	50.61	15.84	33.55
Spectrum 2	52.90	14.43	32.67
Spectrum 3	52.86	15.18	31.96
Spectrum 4	52.56	15.38	32.06
Spectrum 5	50.29	16.57	33.14
Mean	**51.84**	**15.48**	**32.68**

Fig. S1 Energy-dispersive X-ray (EDX) results. a Atomically resolved elemental mappings of FGT, including a HAADF-STEM image along the [100] zone axis and

individual distribution of Fe, Ga and Te elements. **b-d** EDX spectrum of five regions in FGT. A mean atomic percentage ratio of Fe: Ga: Te = 51.84: 15.48: 32.68 was acquired, which gived a chemical composition of $Fe_{3.35}Ga_{1}Te_{2.11}$.

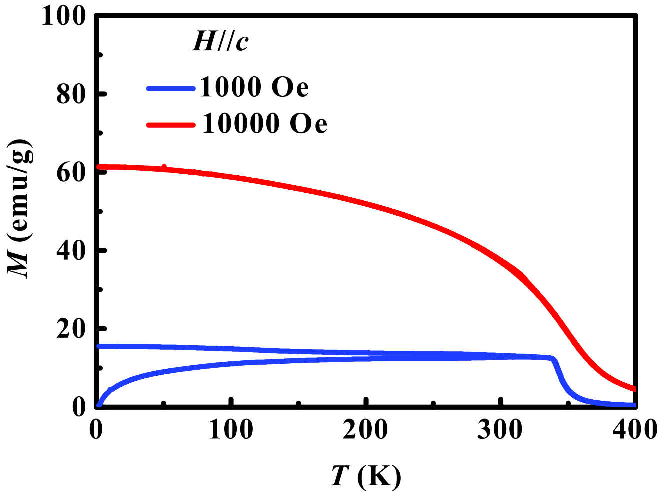

Fig. S2 Temperature dependence of magnetization curves with a magnetic field of 1000 and 10000 Oe.

To help determine the ferromagnetism in FGT crystals, we have done the modified Curies-Weiss fitting via the formula[1]:

$$\frac{H}{M} = \frac{T}{C} - \frac{T_C}{C} \qquad (1)$$

Where C is Curies-Weiss constant, as shown in the set of Fig. S2. The good fitting result yields T_c = 347 K for the bulk FGT sample.

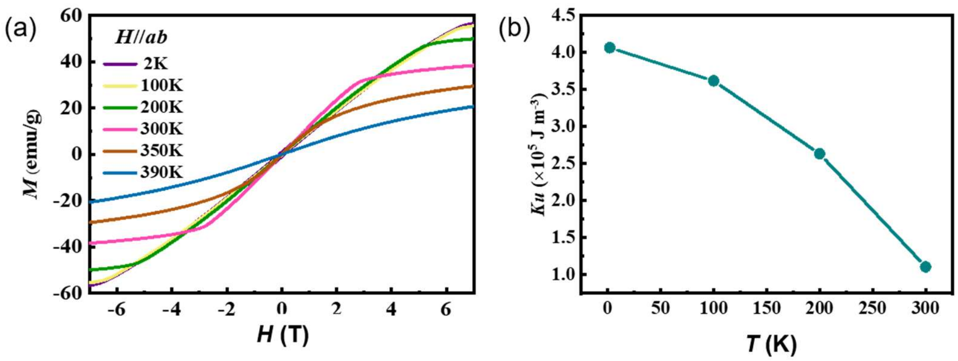

Fig. S3 Temperature-dependent magnetic anisotropy. a M-H curves of FGT bulk crystals at varying temperatures with magnetic fields along the *ab*-plane direction. **b** Temperature dependence of anisotropy constant (K_u) in the temperature range 2-300 K.

The value of K_u was estimated approximated by using the area difference between the out-of-plane and in-plane magnetization curves under each temperature. The positive K_u means the easy-axis magnetization. One can notice that the value of K_u decreases monotonically with the increase of temperature, nevertheless, it still represents a strong out-of-plane anisotropy at room temperature.

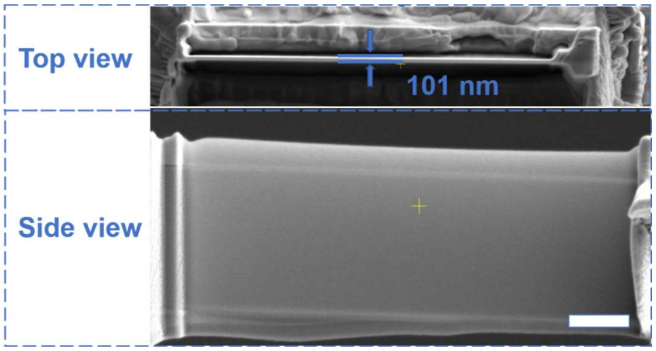

Fig. S4 Thickness measurements of a uniform FGT lamella. SEM images of the top view (upper panel) and side view (bottom panel) of FGT show that the thickness is estimated at 101 nm. The scale bar is 1 μm.

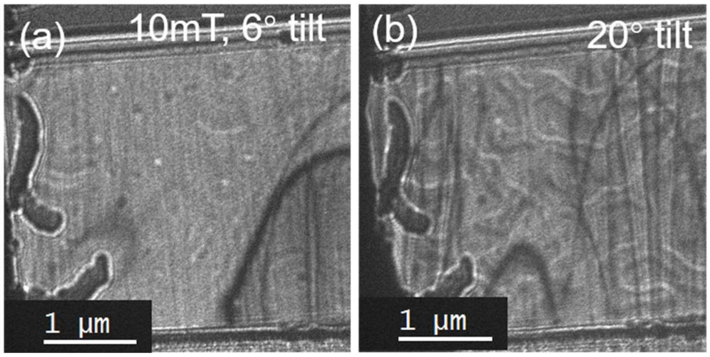

Fig. S5 Tilt-dependent contrast of magnetic domain. a-b Over-focused LTEM images obtained at α =0° (a) and 20° (b) under a magnetic field of 10 mT.

It is obvious that several Bloch skyrmions exist in a lamella tilted at 6° with a magnetic field of 10 mT, as shown in Fig. S5a. More importantly, the hybrid stripes domain is nearly invisible at 6° tilt, which may be attributed to the counteraction between Bloch and Neel contrast. And the hybrid stripes become visual when the lamella is tilted at 20° (see Fig. S5b).

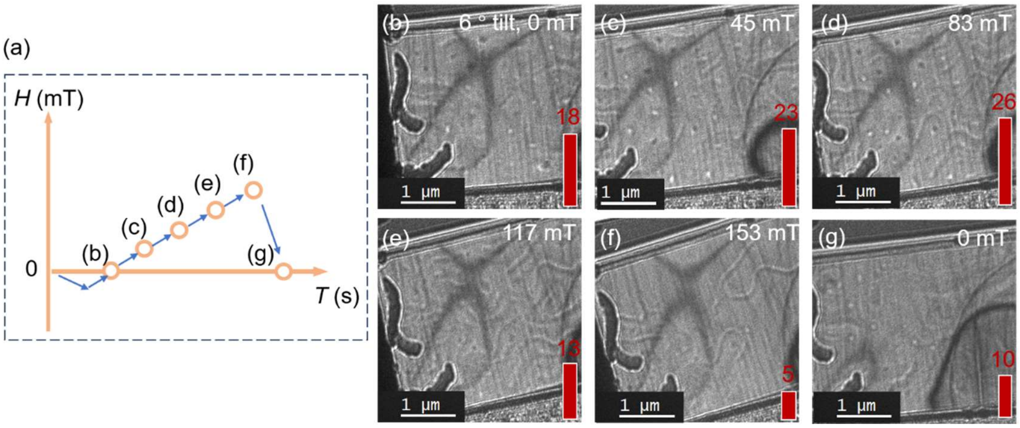

Fig. S6 Evolution of magnetic domain structures in a FGT lamella. a Schematic of the measurement process for b-g**. b-g** Dynamic behaviors of the magnetic domain under

the magnetic field at 6° tilt. The Bloch skyrmions are counted in the set of each figure.

The dynamic behaviors of spin textures under the magnetic field were investigated at 6° tilt, as shown in Fig. S6. It should be noted that the FGT lamella underwent a field-swapping process, with its magnetization history depicted in Fig. S6a. Intriguingly, we observed an initial increase in the number of Bloch skyrmions from 18 to 26 as the magnetic field increased to 83 mT (see Fig. S6b-d). However, upon further increasing the magnetic field, the number of skyrmions began to decrease, as shown in Fig. S6e-g. The reason can be attributed to the fact that the initial state at 0 mT represents a remanent state, indicative of a metastable state rather than a stable one. Consequently, observing a non-monotonic variation in skyrmion numbers is reasonable, which diverges from the trend elaborated upon in Fig. 3a.

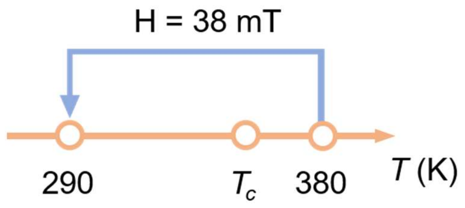

Fig. S7 The schematic diagram for FC procedure. The FGT sample was cooled from 380 K (well above T_c) to 290 K with an out-of-plane magnetic field of 38 mT.

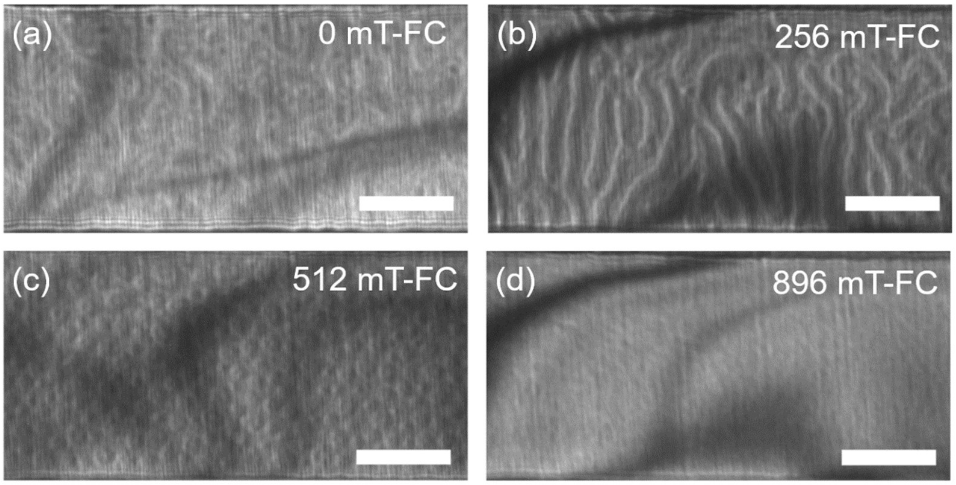

Fig. S8 Magnetic skyrmions in FGT observed at 290 K and zero magnetic field after the FC process with a magnetic field of 0 mT (a), 256 mT (b), 512 mT (c) and 896 mT (d). The tilt angle is 0° (a), and 10° (b-d). The scale bar is 1 μm.

The results of FC-dependent magnetic domain are presented in Fig. S8. It is evident that only Bloch skyrmions and hybrid stripes were observed in FGT after a zero magnetic field cooled (ZFC), as shown in Fig. S8a. With the increment of magnetic field in FC process (see Fig. S8b-c), more hybrid skyrmions were observed and the zero-field hybrid skyrmion lattice were created after the 512 mT-FC process. Notably, increasing the magnetic field further would be not conducive to the formation of skyrmions, and it came in being a ferromagnetic state nearly after the 896 mT-FC process, as presented in Fig. S8d.

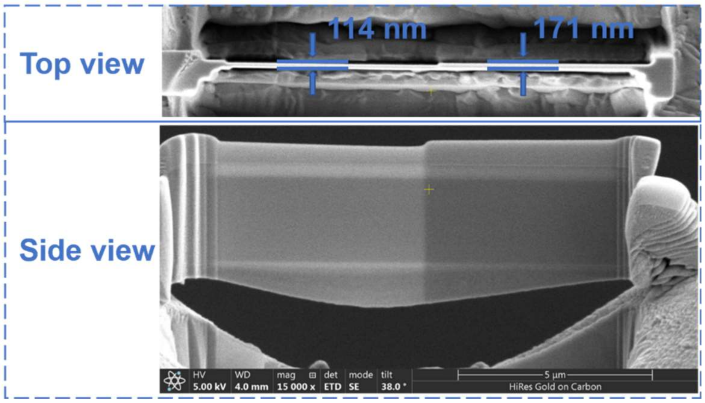

Fig. S9 Thickness measurements of a FGT lamella with two different-thickness

regions. SEM images of the top view (upper panel) and side view (bottom panel) of FGT show that the thickness is estimated at 114 and 171 nm for the thin and thick regions, respectively.

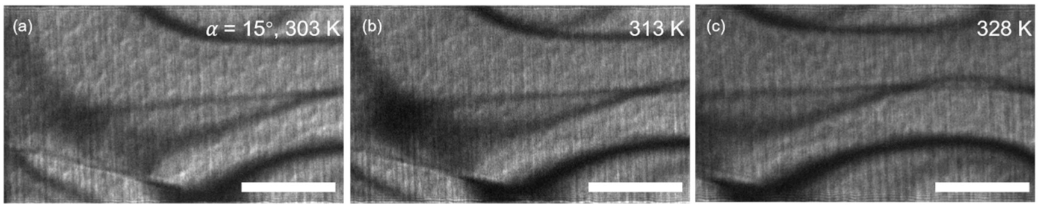

Fig. S10 The thermostability of the magnetic domain in the 114 nm-thick region. a-c Over-focused LTEM images at α =15° at varying temperatures. The scale bar is 1 μm.

One can observe that high-density skyrmions after the FC process remained stable above room temperature, and even were visible at 328 K, which demonstrates the high thermostability of these textures in FGT.

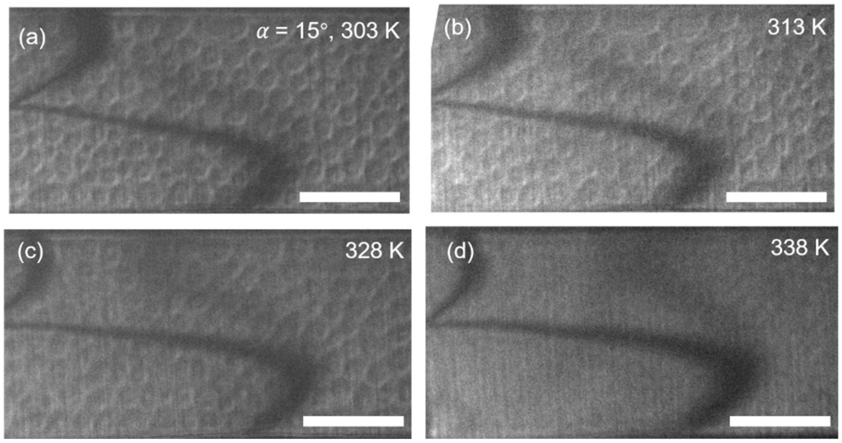

Fig. S11 The thermostability of the magnetic domain in the 171 nm-thick region. a-d Under-focused LTEM images at α =15° at varying temperatures. The scale bar is 1 μm.

In our comparative analysis, skyrmions within the 171 nm-thick region of our sample

exhibited a larger size relative to those in the 114 nm-thick region. This observed size discrepancy is likely due to the enhanced magnetic dipole-dipole interactions present in the thicker sample. Moreover, we noted the persistence of mixed skyrmion phases at a temperature of 328 K. However, these phases appeared to dissipate almost entirely when the temperature was raised to 338 K. Therefore, it is reasonable to expect that skyrmions could withstand higher temperature in samples exceeding 171 nm in thickness, which has not yet been verified further.

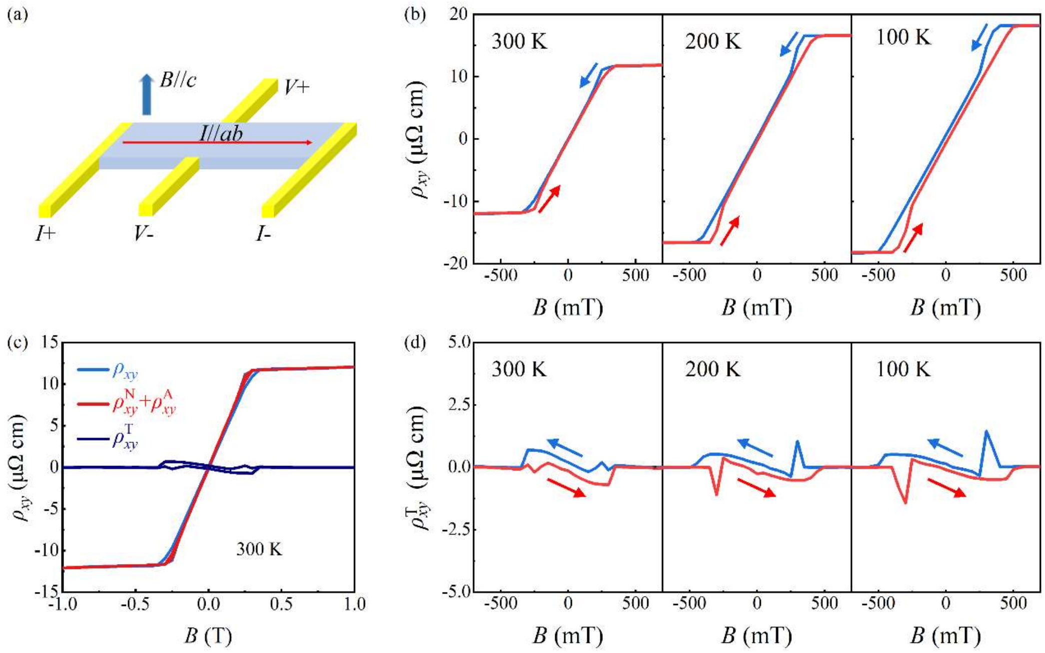

Fig. S12 Transport properties of FGT nanoflake. a Schematic of the FGT Hall device with I // ab plane and B // c axis. b Temperature-dependent Hall resistivity ρ_{xy}. c Representative extraction of topological Hall resistivity $\rho^{T}{}_{xy}$ at 300 K. $\rho^{N}{}_{xy}$ and $\rho^{A}{}_{xy}$ shows the ordinary and anomalous Hall resistivity. d Temperature-dependent topological Hall resistivity $\rho^{T}{}_{xy}$. Blue and red curves were measured with decreasing and increasing magnetic field, respectively.

To investigate the potential THE in FGT, we fabricated a Hall-bar device with a

sample thickness of about 19.58um for the electric transport measurements over the temperature range of 300 -100 K, as shown in Fig. S12. Fig. S12a shows the schematic of the FGT Hall device with I // ab plane and B // c axis. The results show that there is a discrepancy at the low-field region in magnetic hysteresis of Hall resistivity ρ_{xy}, demonstrating the presence of a pronounced THE component (see Fig. S12b). By linearly fitting the ρ_{xy}, we obtained the field dependent extracted topological Hall resistivity ρ^{T}_{xy} at various temperature (see Fig. S12c-d). It is evident that the THE signals are remarkable at 100 K and the maximum value of ρ^{T}_{xy} reach to 1.46 μΩ cm. With the increment of temperature, the ρ^{T}_{xy} reduces monotonically but even apparent at 300 K with a maximum value of 0.73 μΩ cm. The existence of such broad-temperature THE signals shows good agreement with the observations of high-thermostability topological skyrmions by LTEM.

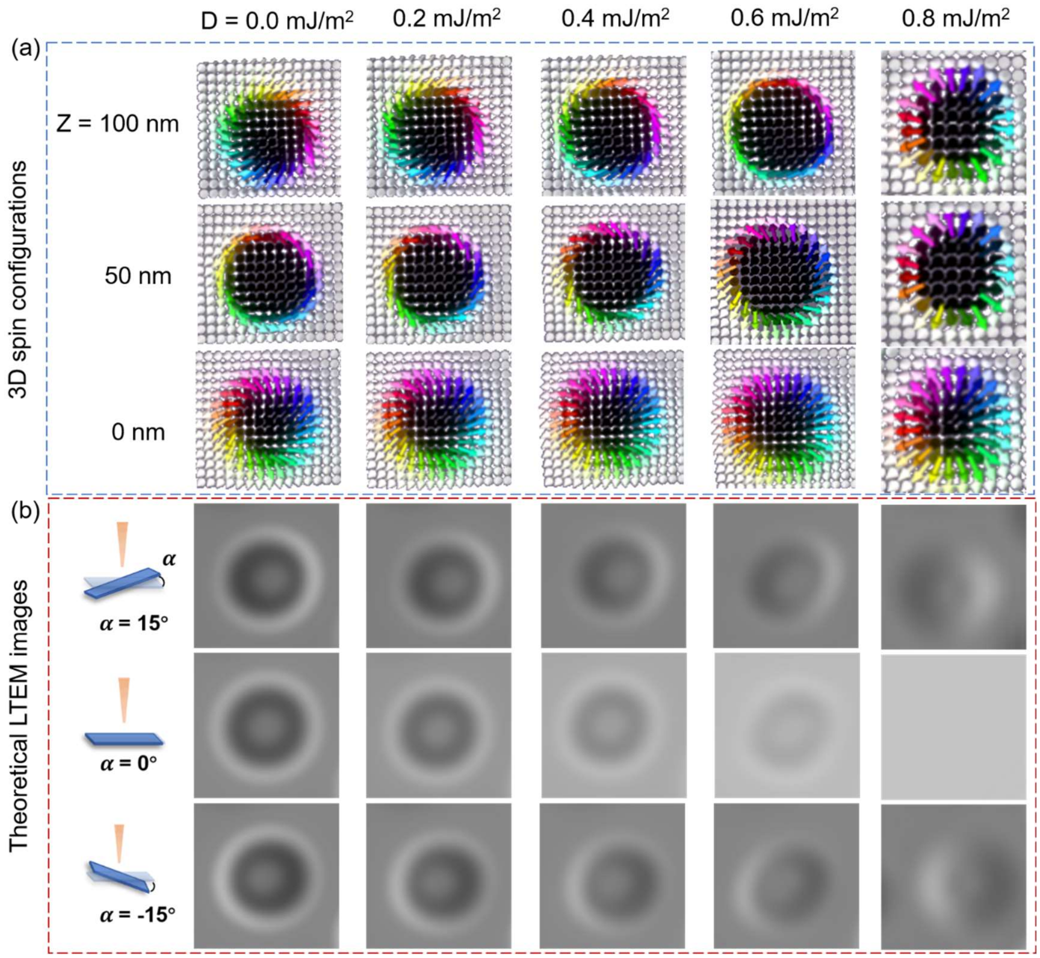

Fig. S13 Micromagnetic simulations and theoretical LTEM images. a Simulated magnetic transformations of 3D spin configurations between Bloch to hybrid to Neel skyrmions with increasing DMI strength. **b** Corresponding theoretical LTEM images obtained at different tilted angles.

Fig. S13a illustrates the DMI-dependent 3D spin configurations, revealing a transformation from Bloch to hybrid, and eventually to Neel type as the DMI increases. This is further substantiated by corresponding theoretical LTEM images, which display distinct magnetic contrasts correlating with these transitions, as shown in Fig. S13b. Additionally, it is necessary to mention that skyrmions with D = 0.0 mJ/m^2 are catergorised as Bloch skyrmions, and those with D =0.8 mJ/m^2 as Neel skyrmions. Skyrmions with D values ranging between 0 and 0.8 mJ/m^2 should be classified into hybrid skyrmions. The main difference between these hybrid skyrmions is their varying degrees of hybridization, i.e., different proportions of Bloch and Neel components.

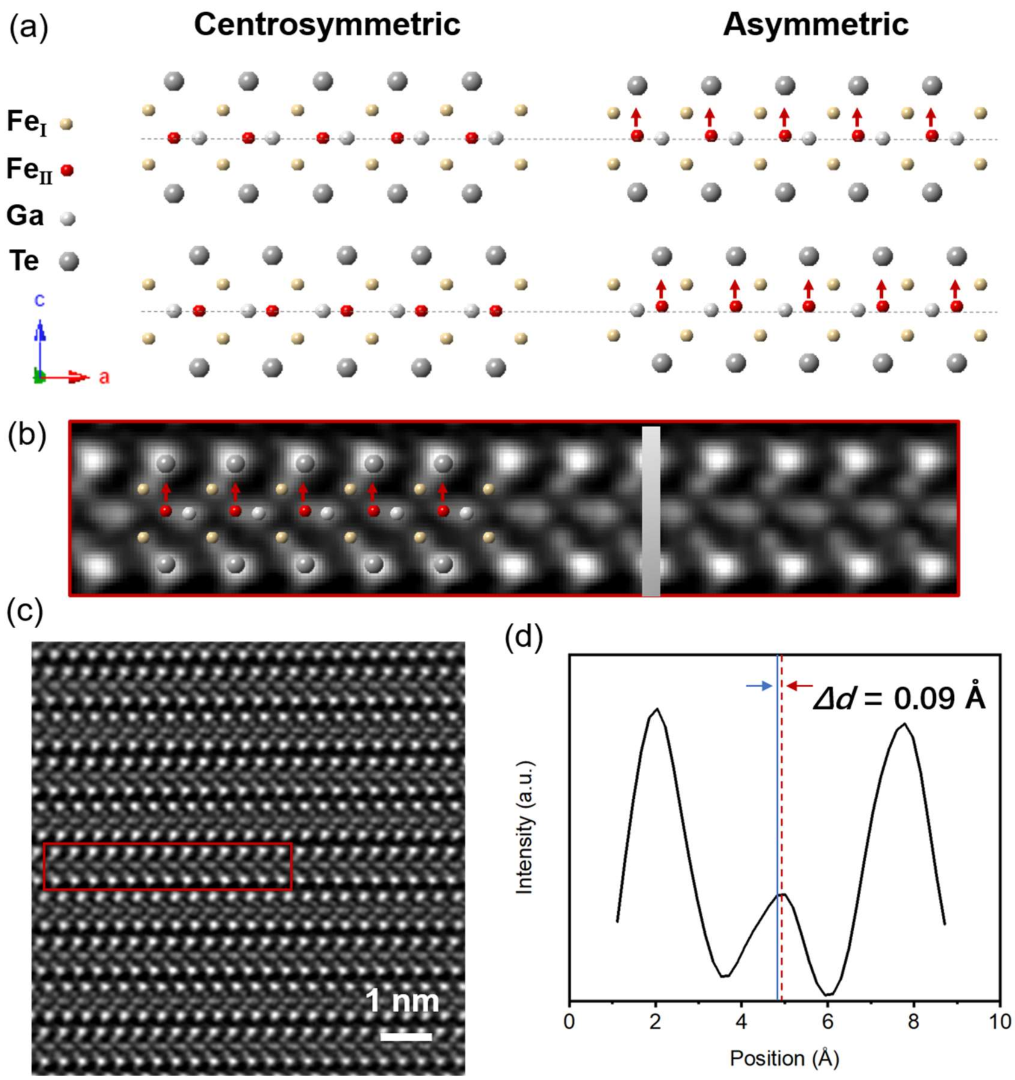

Fig. S14 The analysis of crystal structure of FGT. **a** Schematic of atom arrangements in centrosymmetric and asymmetric FGT along *c* axis. **b** Observation of Fe_{II} atom deviation. The region is selected from a atomic-resolution HAADF-STEM image (c). **d** Line profie of the image intensity shown in (b). The blue and red lines represent the central position of two Te atoms and Fe_{II} atom, and their distance is estimated at 0.09 Å, respectively.

Recent studies have asserted that the displacement deviation of Fe_{II} atom in FGT can

induce the DMI,[2,3] as illustrated in Fig. S14a. To validate the hypothesis, we have further acquired an improved HAADF-STEM image, as depicted in Fig. S14b-c. Subsequently, for a quantitative determination of the displacement of the Fe_{II} atom, we performed a vertical integration of the corresponding imaging intensity line profile (Fig. S14d). By referencing the midpoint of the two Te atoms, the deviation of the Fe_{II} atom towards the *c*-axis was determined to be –0.09 Å."

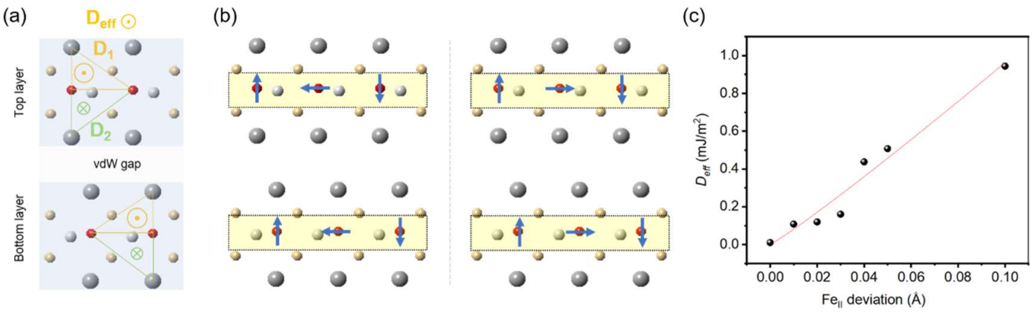

Fig. S15 The results of first-principles calculations. a Schematic of DMI in FGT. The orange arrow D_1 shows the direction of DMI vector induced by Fe_{II} and top Te atoms, while the green arrow D_2 represents the opposite direction of DMI vector induced by Fe_{II} and bottom Te atoms. D_1 is not equal to D_2, leading to a net DMI D_{eff}. **b** Spin configurations of counter-clockwise (CCW) (left column) and clockwise (CW) (right column). **c** Fe_{II} atom deviation-dependent D obtained by first-principles calculations.

To further investigated the relationship between the deviation of the Fe_{II} atom and DMI constant *D*, density functional theory (DFT)-based first-principles calculations were employed, as depicted in Fig. S15. The calculation of the DMI vector occurred in two steps. Initially, structural relaxations were performed with a fixed δ(Fe) using Gaussian smearing until the forces diminished to less than 0.001 eV/Å. Subsequently, spin-orbit coupling was integrated into the calculation, and the system's total energy was determined based on the spin configuration, as illustrated in Fig. S15b. The parameter *d* was determined as (E_{CCW}-E_{CW})/12, where E_{CCW} denotes the energy of the counter-clockwise configuration and E_{CW} denotes the energy of the clockwise configuration.[4] The DMI constant *D* was then derived using the equation $D =$

$3\sqrt{2d}/(N_F a^2)$, where NF represents the number of atomic layers, a is the lattice constant, and *d* represents the DMI strength. In the second step, the EDIFF parameter was set to 10^-6 eV, and the tetrahedron method was employed to obtain an accurate total energy. The results are depicted in Fig. S15c. It is clear that the absence of Fe_{II} atom deviation yields $D = 0$ mJ/m^2, indicating an ideally centrosymmetric crystal structure of FGT. As the Fe_{II} atom deviation increases, the value of D increases monotonously and reaches 0.94 mJ/m^2 at a 0.1 Å deviation. This value might be slightly larger than the actual one in our experiments, which is attributed to the omission of some realistic factors such as temperature and nonuniformity due to DFT's limitations. It should be highlighted that the estimated value of D (< 0.6 mJ/m^2) obtained through micromagnetic simulations also aligns within the range of DFT-calculated values, underscoring the credibility of our findings.

References

1 Mugiraneza, S. & Hallas, A. M. Tutorial: a beginner's guide to interpreting magnetic susceptibility data with the Curie-Weiss law. *Commun. Phys.* **5**, 95 (2022).

2 Li, Z. *et al.* Room-temperature sub-100 nm Néel-type skyrmions in non-stoichiometric van der Waals ferromagnet Fe3-x GaTe2 with ultrafast laser writability. *Nat. Commun.* **15**, 1017 (2024).

3 Liu, C. *et al.* Controllable Skyrmionic Phase Transition between Néel Skyrmions and Bloch Skyrmionic Bubbles in van der Waals Ferromagnet Fe3-δGeTe2. *Adv. Sci.* **10**, 2303443 (2023).

4 Yang, H., Thiaville, A., Rohart, S., Fert, A. & Chshiev, M. Anatomy of dzyaloshinskii-moriya interaction at Co/Pt interfaces. *Phys. Rev. Lett.* **115**, 267210 (2015).
